# Disulfiram Attenuates Adenine‐Induced Renal Fibrosis by Modulating Inflammatory and Immunometabolic Responses

**DOI:** 10.1096/fj.202601271RR

**Published:** 2026-09-17

**Authors:** Saori Kin, Yuji Oe, Shun Ishigaki, Ichiro Fujio, Mariko Miyazaki, Tetsuhiro Tanaka

**Affiliations:** ^1^ Department of Nephrology, Graduate School of Medicine Tohoku University Sendai Japan; ^2^ School of Medicine Tohoku University Sendai Japan

**Keywords:** cellular senescence, fatty acid oxidation, inflammasome, pyroptosis, renal fibrosis

## Abstract

Chronic kidney disease (CKD) is partly driven by excessive innate immune activation, which contributes to progressive renal injury and fibrosis. Disulfiram (DSF), an approved treatment for alcohol dependence, has recently emerged as an inhibitor of innate immune responses such as pyroptosis. In this study, we investigated whether DSF attenuates kidney injury in adenine‐induced nephropathy and explored the underlying mechanisms. DSF treatment markedly ameliorated kidney injury in adenine‐induced nephropathy, accompanied by suppression of inflammatory cell infiltration and inflammasome‐ and pyroptosis‐related markers. RNA sequencing revealed restoration of disrupted fatty acid metabolism in the kidneys following DSF treatment. Furthermore, DSF alleviated DNA damage, cellular senescence, and fibrosis. Importantly, activation of gasdermin D, a central executor of pyroptosis, was closely associated with disease severity in human CKD biopsy samples, supporting the clinical relevance of this pathway. Collectively, these findings suggest a potential role for DSF in modulating inflammatory and immunometabolic pathways in CKD.

## Graphical abstract

Chronic kidney disease (CKD) is characterized by persistent inflammation that drives progressive renal injury and fibrosis. In this study, we demonstrate that disulfiram markedly attenuates kidney injury and fibrosis in adenine‐induced nephropathy by suppressing innate immune responses and reducing DNA damage and cellular senescence. These findings support the potential repurposing of disulfiram as a novel innate immune modulator for the treatment of CKD.

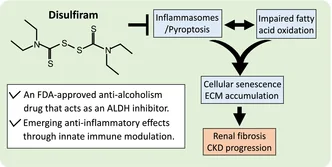

## 1 Introduction

The burden of chronic kidney disease (CKD) is increasing globally, and is strongly linked to the development of end‐stage renal disease, cardiovascular complications, and mortality [1]. Despite recent therapeutic advances, including the use of renin–angiotensin system inhibitors, mineralocorticoid receptor antagonists, and sodium–glucose cotransporter 2 inhibitors, CKD progression remains incompletely controlled in a significant proportion of patients [2, 3]. Therefore, identification of novel therapeutic targets to prevent CKD progression is required.

Accumulating evidence indicates that CKD is characterized by persistent, low‐grade chronic inflammation [4, 5]. In particular, dysregulation of innate immune pathways, including NLRP3 inflammasome activation, plays a critical role in renal fibrosis and disease progression [6, 7]. Recent studies have revealed the pathological role of pyroptosis, a form of programmed cell death downstream of innate immune signaling, further implicating these pathways in CKD pathogenesis [8]. Consistent with the concept of immunothrombosis, coagulation is increasingly recognized as a part of the innate immune response, and our previous work demonstrated its contribution to CKD progression [9, 10, 11].

Disulfiram (DSF) is an aldehyde dehydrogenase (ALDH) inhibitor that has been used clinically for decades to treat alcohol use disorder [12]. Although originally developed for this purpose, recent studies have shown that DSF exerts previously unknown immunomodulatory effects [13]. DSF inhibits gasdermin D (GSDMD)‐mediated pore formation, thereby suppressing pyroptosis and pro‐inflammatory cytokine release. Additionally, GSDMD inhibits the activation of toll‐like receptor 4 (TLR4) signaling or the NLRP3 inflammasome [14, 15, 16]. By modulating these innate immune pathways, DSF has attracted attention as a potential therapeutic agent in experimental models of acute kidney injury (AKI) and CKD [17, 18, 19].

This study aimed to demonstrate the therapeutic effects of DSF in an adenine‐induced renal fibrosis model and characterize its modulatory roles in renal immunometabolism during kidney injury. Additionally, we examined the association between *GSDMD* expression and CKD severity using public databases and renal biopsy samples.

## 2 Methods

### 2.1 Animal Study

All animal procedures complied with the guidelines of Tohoku University and the ARRIVE recommendations. The study protocols were approved by the Institutional Animal Care and Use Committee of Tohoku University (approval no. 2021MED‐040‐03). Eight‐week‐old male C57BL/6J mice were purchased from CLEA Japan (Tokyo, Japan). In protocol 1, the mice were fed an adenine‐containing diet supplemented with DSF (0.1% w/w; LKT Laboratories, St. Paul, MN, USA) for 2 weeks. Adenine concentration was 0.25% during the first week, which decreased to 0.2% during the second week. The adenine‐containing diet was based on an MF diet (Oriental Yeast, Tokyo, Japan). In protocol 2, the mice were fed a 0.25% adenine‐containing diet during the first week, followed by a 0.2% adenine‐containing diet during the second week. Following the induction of renal injury, mice were placed on a diet supplemented with 0.1% (w/w) DSF for an additional 4 weeks. Mice were housed in groups of two per cage, and food consumption was measured every 2–3 days. Under these conditions, the estimated DSF intake was approximately 100 mg/kg/day.

### 2.2 Renal Function Assay

Blood urea nitrogen (BUN) was quantified using a chromogenic assay (Arbor Assays, Ann Arbor, MI, USA), whereas plasma cystatin C concentrations were determined with a commercially available ELISA kit (Assaypro, Saint Charles, MO, USA). All assays were conducted in accordance with the manufacturers' protocols.

### 2.3 Real‐Time Quantitative Polymerase Chain Reaction (PCR)

Renal tissues were homogenized using a bead‐based homogenizer (TOMY; Tokyo, Japan). Total RNA was subsequently purified using the RNeasy Plus Mini Kit (Qiagen, Germantown, MD, USA). Complementary DNA was synthesized using the iScript Advanced cDNA Synthesis kit (Bio‐Rad, Hercules, CA, USA), following the manufacturer's instructions. Quantitative real‐time PCR was performed using the KAPA SYBR Fast qPCR kit (KAPA Biosystems, Wilmington, MA, USA). Thermal cycling conditions comprised an initial denaturation at 98°C for 3 min, followed by 40 amplification cycles of denaturation at 95°C for 5 s and annealing/extension at 60°C for 10 s. *Hprt* served as the housekeeping gene for normalization. Details of the primer sequences are shown in Table S1.

### 2.4 RNA Sequencing (RNA‐Seq) Analysis

RNA‐seq libraries were prepared and sequenced on a NovaSeq X Plus platform by Azenta Life Sciences (Tokyo, Japan). Raw reads were quality‐filtered and trimmed using fastp. Transcript quantification was performed with Salmon against the mouse reference transcriptome (GRCm39), and gene‐level counts were generated using tximport. Differential expression analysis was conducted using edgeR after filtering lowly expressed genes with filterByExpr. Genes with |log_2_ fold change| > 1 and FDR < 0.05 were considered differentially expressed. All analyses were performed using the OlvTools platform (https://olvtools.com/; BxINFO, Tokyo, Japan). Gene set enrichment analysis was performed using the ShinyGO 0.85 web tool with an FDR cutoff of < 0.05, displaying the top 10 Kyoto Encyclopedia of Genes and Genomes (KEGG) pathways selected by FDR and ranked according to fold enrichment (http://bioinformatics.sdstate.edu/go/) [20]. Heatmaps were generated using the Heatmapper2 web tool (http://www.heatmapper2.ca/) [21]. The RNA‐seq data generated in this study have been deposited in the DNA Data Bank of Japan (DDBJ) under BioProject accession number PRJDB42838.

### 2.5 Immunohistochemistry in Animal Study

Renal tissues were fixed in 4% paraformaldehyde and embedded in paraffin. Paraffin blocks were sectioned into 3‐μm‐thick slices for immunohistochemical analysis. The following primary antibodies were used: rabbit antihuman CD68 (1:2000, #ab125212, Abcam, Cambridge, UK), rabbit antihuman myeloperoxidase (MPO) polyclonal antibody (1:5000, #22225‐1‐AP, Proteintech, Rosemont, IL, USA), rabbit antihuman CPT1A polyclonal antibody (1:2000, #15184‐1‐AP, Proteintech), rabbit antihuman phospho‐histone H2A.X monoclonal antibody (1:1000, #9718, Cell Signaling Technology, Danvers, MA, USA), and rabbit anti‐collagen Type IV polyclonal antibody (1:1000, #AB756P, EMD Millipore Corporation, Temecula, CA, USA). Heat‐induced antigen retrieval was performed using sodium citrate buffer (pH 9.0) for CD68 and sodium citrate buffer (pH 6.0) for phospho‐histone H2A.X. Dako Target Retrieval Solution (Dako, Glostrup, Denmark) was used for antigen retrieval from other antigens. N‐Histofine Simple Stain kits (Nichirei Biosciences, Tokyo, Japan) were used for secondary antibodies. Signal detection was achieved using the N‐Histofine Simple Stain kit (Nichirei Biosciences, Tokyo, Japan). Sections lacking primary antibody incubation served as negative controls.

### 2.6 Histological Evaluation in Animal Study

Kidney sections were subjected to Elastica–Masson trichrome and hematoxylin–eosin staining to examine tubulointerstitial lesions. Tubular damage was evaluated semi‐quantitatively according to the proportion of injured tubules within the field. Scores ranged from 0 to 5, corresponding to none, < 10%, 10%–25%, 26%–50%, 51%–75%, and 76%–100% involvement. Areas positive for CD68, MPO, CPT1A, and collagen Type IV were quantified and expressed as a ratio to the total renal cortical area using ImageJ software (version 1.54; National Institutes of Health, Bethesda, MD, USA; https://imagej.nih.gov/ij). For these analyses, three consecutive fields of the renal cortex per section were examined at ×100 magnification. The number of phospho‐histone H2A.X‐positive cells was counted per high‐power field in 10 nonoverlapping fields per sample. All histological evaluations were performed in a blinded manner.

### 2.7 Evaluation of Cleaved GSDMD Expression in Renal Biopsy Specimens

The protocol received approval from the Institutional Review Board of Tohoku University Hospital (No. 2025‐1‐589) and was carried out in accordance with the Declaration of Helsinki. Informed consent was obtained using an opt‐out method, and information regarding the study was disclosed on the institution's website, allowing patients the opportunity to decline participation. To evaluate the association between renal expression of cleaved GSDMD and histological and clinical disease severity, all patients diagnosed with IgA nephropathy after undergoing renal biopsy in our department between August 2023 and July 2024 were included in the analysis. Finally, 27 patients were included in the analysis. Renal tissues were fixed with ethanol, embedded in paraffin, and sectioned at 2 μm thickness. Sections were incubated with rabbit anti‐human cleaved GSDMD antibody (1:500; #36425; Cell Signaling Technology, Danvers, MA, USA). Heat‐induced antigen retrieval was performed using a sodium citrate buffer (pH 6.0). Immunoreactivity was visualized using an EnVision FLEX HRP detection system (Dako, Glostrup, Denmark). For histological evaluation, all the tissue sections were examined. Samples were defined as “positive” if any positive staining was observed in the section, whereas samples were classified as “negative” when no positive staining was detected in any of the observed areas. The analysis was carried out in a blinded fashion.

### 2.8 Human CKD Transcriptomics Datasets

Nephroseq v4 (https://www.nephroseq.org/) and the Saint‐Mézard transplant dataset were used to analyze *GSDMD* expression and its correlation with estimated glomerular filtration (eGFR) and the expression of other genes [22].

### 2.9 Statistical Analysis

All statistical analyses were conducted with JMP Pro 15 software (SAS Institute, Cary, NC, USA). Differences were considered statistically significant at *p* < 0.05. Normality was assessed using the Shapiro–Wilk test. In animal experiments, outliers were identified using the 1.5 × interquartile range (IQR) rule. For comparisons between two groups, Student's *t*‐test was used for normally distributed data, whereas the Wilcoxon rank‐sum test was applied for non‐normally distributed data. For comparisons among multiple groups, one‐way analysis of variance (ANOVA) followed by the Tukey–Kramer post hoc test was performed for normally distributed data. When the data were not normally distributed, either log transformation was applied prior to parametric testing or the Kruskal–Wallis test followed by the Steel–Dwass post hoc test was used. In the human study, parametric and nonparametric variables were compared between two groups using the Student's *t*‐test and Wilcoxon rank‐sum test, respectively, while categorical variables were compared using the *χ* ^2^ test. Correlation analyses were performed using Spearman's rank correlation coefficient. Data are presented as mean ± standard error of the mean or as violin plots.

## 3 Results

### 3.1 DSF Alleviates Renal Dysfunction and Histological Injury Scores in Adenine‐Induced Nephropathy

**FIGURE 1:**
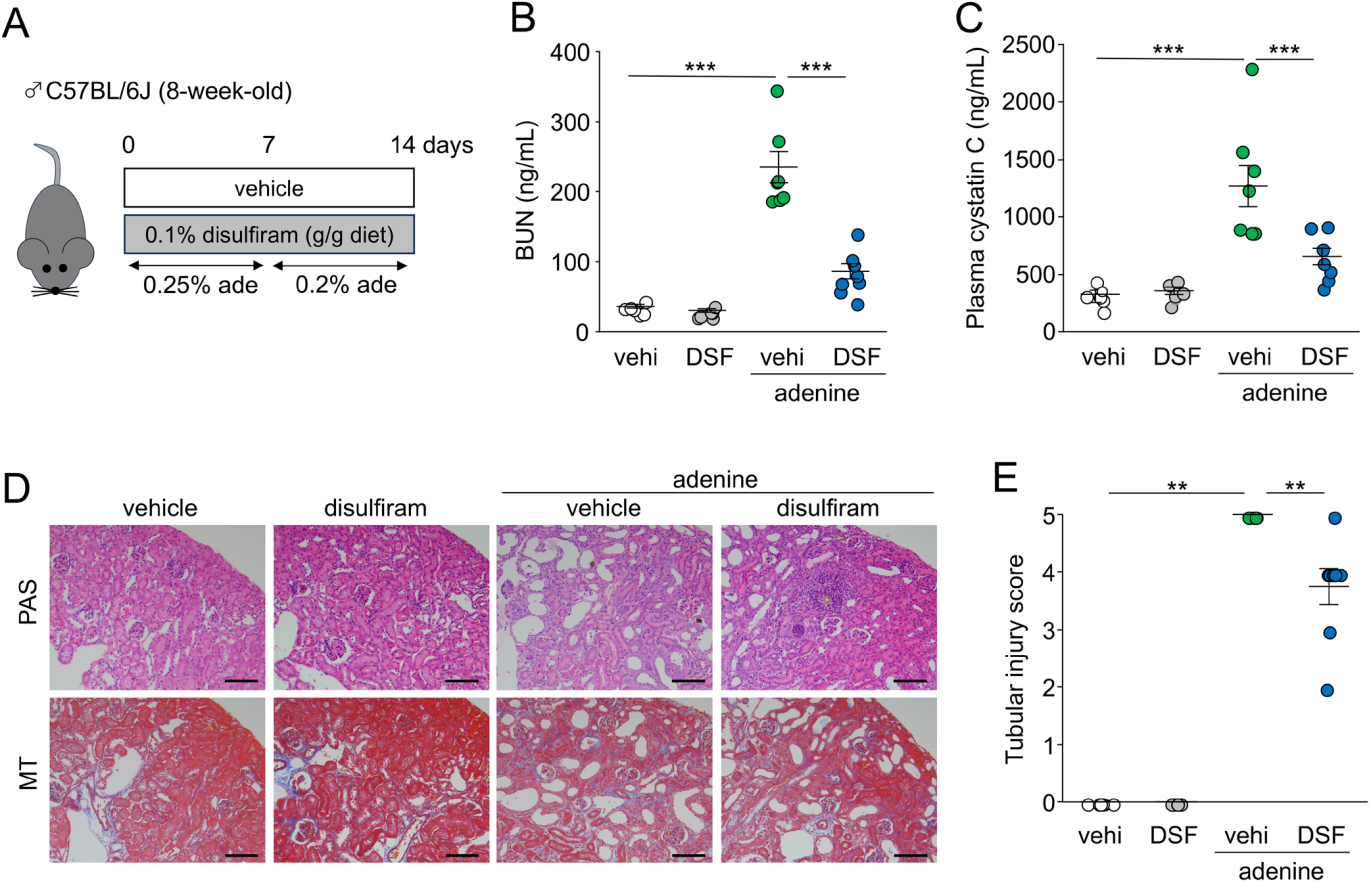
DSF alleviates renal dysfunction and tubular injury in adenine‐induced nephropathy. (A) Experimental protocol. Plasma levels of blood urea nitrogen (BUN) (B) and cystatin C (C). (D) Representative photomicrographs of periodic acid–Schiff (PAS) and Masson's trichrome (MT) staining of the kidney. Scale bar = 100 μm. (E) Comparison of tubular injury scores. ade, adenine; DSF, disulfiram; vehi, vehicle. Data are presented as mean ± standard error of the mean. *n* = 6–8. **p < 0.01, ***p < 0.001.

To investigate the effects of DSF on adenine‐induced nephropathy, the mice were divided into four groups: control, control treated with DSF, adenine, and adenine treated with DSF (Table S2). The experimental protocol is shown in Figure 1A. Adenine administration resulted in significant increases in BUN and plasma cystatin C levels compared with those in the control mice. These renal dysfunction indices were significantly attenuated by DSF treatment in adenine‐fed mice. In contrast, DSF administration alone did not affect renal function in control mice (Figure 1B,C). Food intake decreased, likely due to uremia induced by adenine administration; however, it showed improvement, particularly during the later period, with DSF treatment (Table S2). Histological analysis further demonstrated marked tubular dilation and injury in the kidneys of adenine‐fed mice. These pathological changes were substantially ameliorated by DSF treatment (Figure 1D). We further evaluated tubular injury scores and found that DSF treatment significantly reduced tubular injury in adenine‐induced nephropathy (Figure 1E).

### 3.2 DSF Reduces Inflammasome and Pyroptosis Signaling in Adenine‐Induced Kidney Injury

**FIGURE 2:**
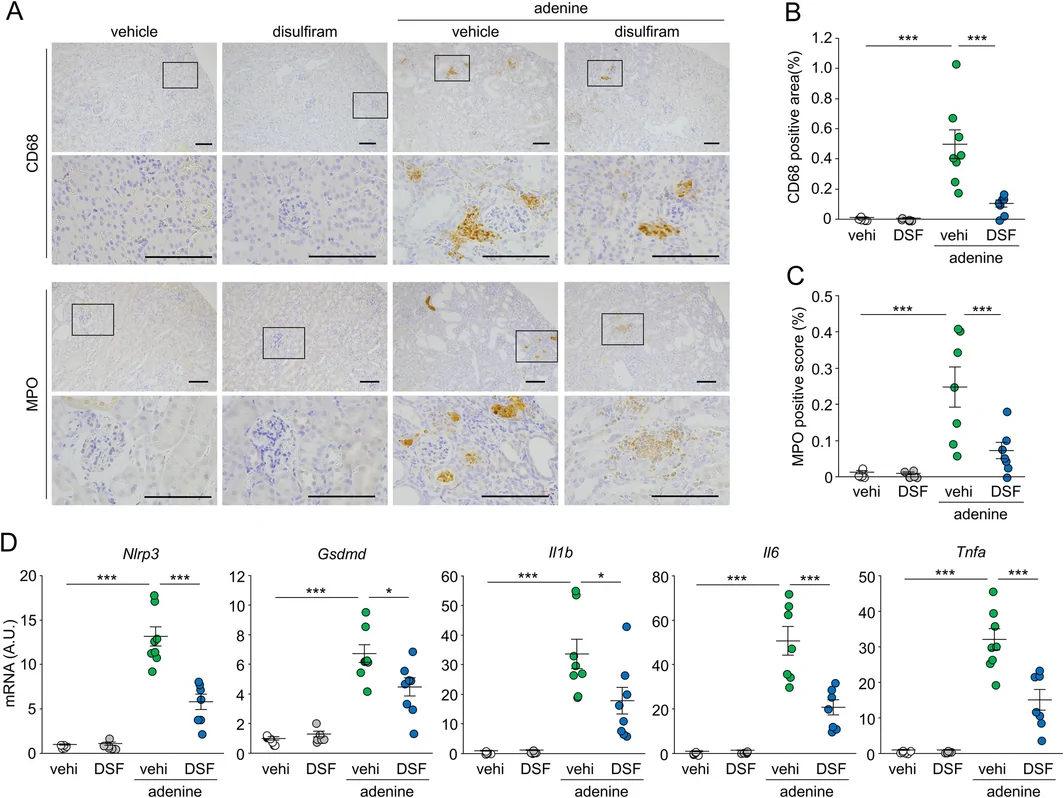
DSF reduces inflammatory cell infiltration and inflammasome and pyroptosis signaling in adenine‐induced kidney injury. (A) Representative photomicrographs of CD68 and myeloperoxidase (MPO) staining of the kidney. The boxed area in the low‐magnification image (upper panel) indicates the region shown at higher magnification in the lower panel. Scale bar = 100 μm. Comparison of CD68‐positive (B) and MPO‐positive (C) areas. (D) Gene expression of *Nlrp3*, *Gsdmd*, *Il1b*, *Il6*, and *Tnfa* in the kidney. A.U., arbitrary units; DSF, disulfiram; vehi, vehicle. Data are presented as mean ± standard error of the mean. *n* = 6–8. *p < 0.05, ***p < 0.001.

Next, we examined the effects of DSF on renal inflammation and inflammasome/pyroptosis‐related pathways. Representative photomicrographs of CD68 and MPO immunostaining in the kidney are shown in Figure 2A. Quantitative analysis demonstrated that the CD68‐positive area and MPO‐positive scores were significantly higher in the adenine group than those in the control group. However, these increases were significantly reduced by DSF treatment (Figure 2B,C). Consistent with the histological findings, adenine administration markedly upregulated renal mRNA expression levels of inflammasome/pyroptosis‐related genes, including *Nlrp3*, *Gsdmd*, and *Il1b*, and DSF treatment significantly attenuated this upregulation (Figure 2D). Similarly, the expression levels of pro‐inflammatory cytokines, such as *Il6* and *Tnfa*, which were elevated in adenine‐induced nephropathy, were significantly reduced by DSF administration (Figure 2D).

### 3.3 Renal RNA‐Seq Identify Attenuation of Fatty Acid Oxidation (FAO)‐Related Pathway by DSF in Adenine‐Induced Nephropathy

**FIGURE 3:**
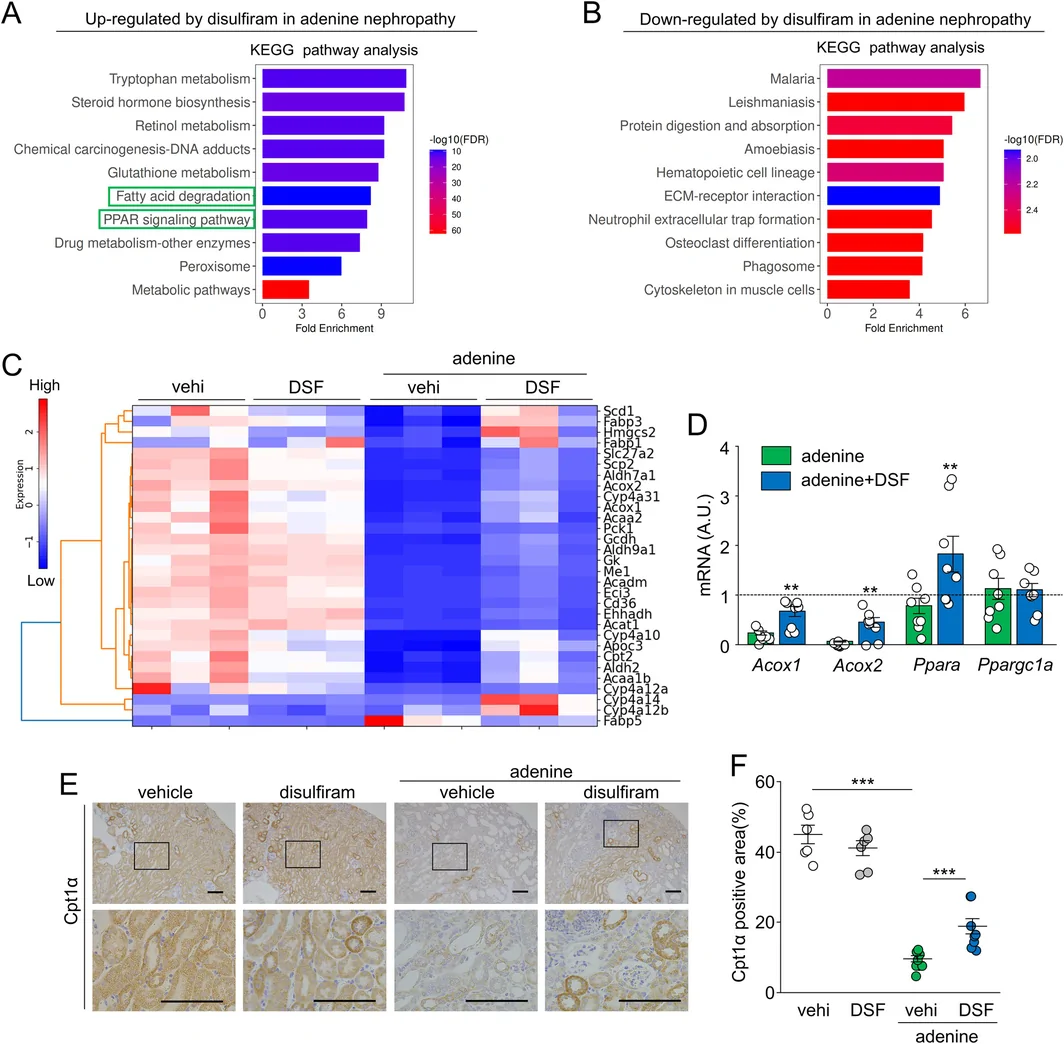
DSF modulates fatty acid metabolism–related pathways in adenine‐induced nephropathy. (A, B) KEGG enrichment analysis of differentially expressed genes between vehicle and DSF treatment in adenine‐induced nephropathy. The top 10 up‐ or down‐regulated pathways are shown. Color scales are shown separately because upregulated and downregulated pathway enrichment analyses were performed independently. (C) Heatmap of differentially expressed genes related to fatty acid metabolism. (D) Expression levels of *Acox1, Acox2, Ppara*, and *Ppargc1a* mRNA in the kidney. (E) Photomicrographs of immunohistochemistry for Cpt1α. The boxed area in the low‐magnification image (upper panel) indicates the region shown at higher magnification in the lower panel. Scale bar = 100 μm. (F) Comparison of Cpt1α‐positive areas in the kidneys. A.U., arbitrary units; DSF, disulfiram; KEGG, Kyoto Encyclopedia of Genes and Genomes; vehi, vehicle. Data are presented as mean ± standard error of the mean. *n* = 6–8. **p < 0.01, ***p < 0.001.

To gain further insight, RNA‐seq and pathway enrichment analyses were performed on the kidneys. Comparison between the control and adenine‐induced nephropathy groups revealed 6166 differentially expressed genes (DEGs; 2693 downregulated and 3473 upregulated). The KEGG pathway enrichment analysis identified downregulated and upregulated pathways (Figure S1A–C). Only six DEGs were detected between the vehicle‐ and DSF‐treated normal diet‐fed mice (data not shown). More importantly, a comparison between the vehicle and DSF treatment in adenine‐induced nephropathy revealed 917 DEGs (309 downregulated and 608 upregulated; Figure S2A,B). The KEGG pathway analysis revealed that the genes upregulated by DSF were significantly enriched in metabolic pathways, including tryptophan metabolism, fatty acid degradation, and the peroxisome proliferator‐activated receptor (PPAR) signaling pathway (Figure 3A). In contrast, the genes downregulated by DSF were enriched in pathways associated with inflammation and extracellular matrix remodeling, such as extracellular matrix–receptor interaction and neutrophil extracellular trap formation (Figure 3B). Innate immune sensors are known to alter metabolic responses, such as FAO [23], and heatmap analysis showed genes that were significantly altered by DSF treatment in adenine‐fed mice within the “fatty acid degradation” and “PPAR signaling pathway” gene sets (Figure 3C). Additionally, mRNA expression levels of key fatty acid β‐oxidation‐related genes, including *Acox1*, *Acox2*, and *Ppara*, were significantly increased in adenine‐fed mice treated with DSF compared with those of the vehicle group (Figure 3D). Similarly, immunohistochemical analysis revealed that tubular CPT1α expression, which was reduced in adenine‐induced nephropathy, was restored by DSF treatment (Figure 3E,F).

### 3.4 DSF Alleviates Tubular DNA Damage Response and Cellular Senescence in Adenine‐Induced Nephropathy

**FIGURE 4:**
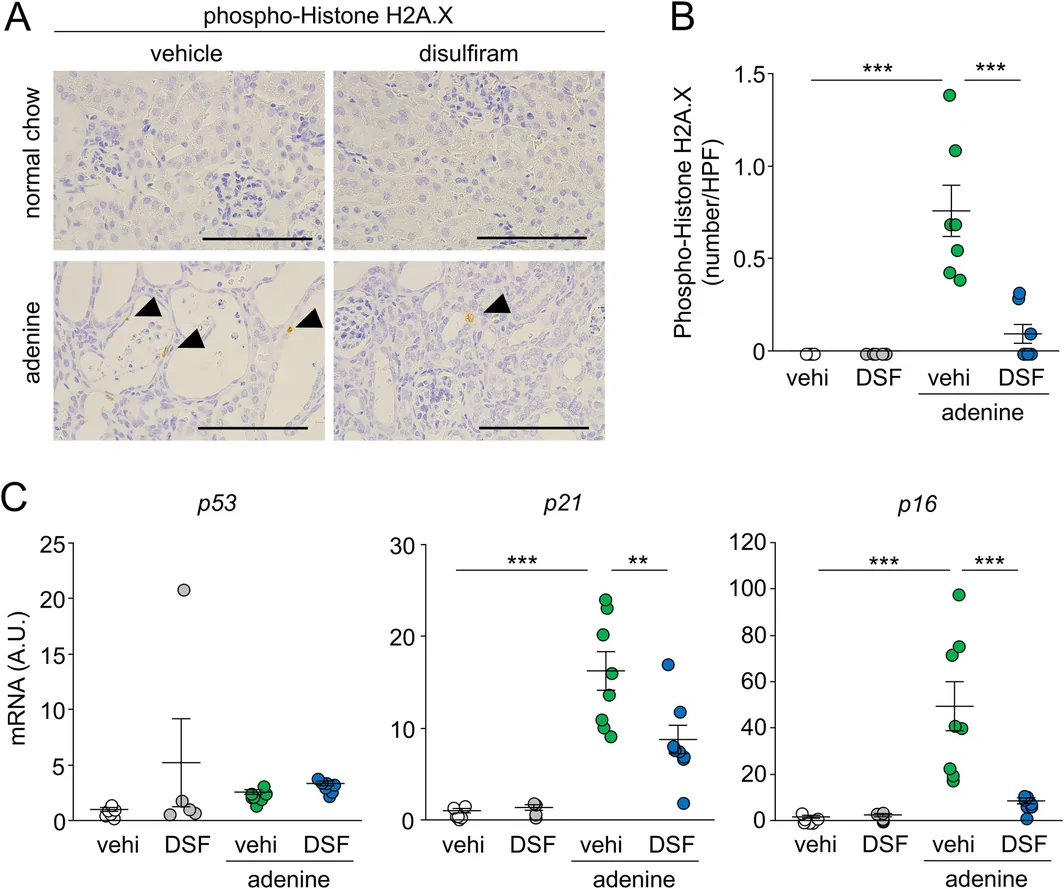
DSF alleviates DNA damage and cellular senescence responses in adenine‐induced kidney injury. (A) Representative photomicrographs of phospho–histone H2A.X staining of the kidney. Arrowheads indicate positive cells. Scale bar = 100 μm. (B) Comparison of the number of phospho–histone H2A.X‐positive cells. (C) Gene expression of *p53*, *p21*, and *p16* in the kidney. A.U., arbitrary units; DSF, disulfiram; vehi, vehicle. Data are presented as mean ± standard error of the mean. *n* = 5–8. **p < 0.01, ***p < 0.001.

Immunohistochemical staining for phosphorylated histone H2A.X (γH2A.X), a marker of DNA double‐strand breaks, revealed a marked increase in γH2A.X‐positive nuclei in the kidneys of adenine‐fed mice compared with that in the controls (Figure 4A). Quantitative analysis demonstrated that the number of γH2A.X‐positive cells was significantly increased in the adenine group, whereas DSF treatment significantly reduced this positivity (Figure 4B). Consistent with the reduced DNA damage signaling, the expression levels of genes related to the DNA damage response and cellular senescence, such as *p21* and *p16*, were markedly elevated in adenine‐fed mice but significantly decreased by DSF treatment (Figure 4C).

### 3.5 DSF Mitigates Renal Fibrotic Responses in Adenine‐Induced Nephropathy

**FIGURE 5:**
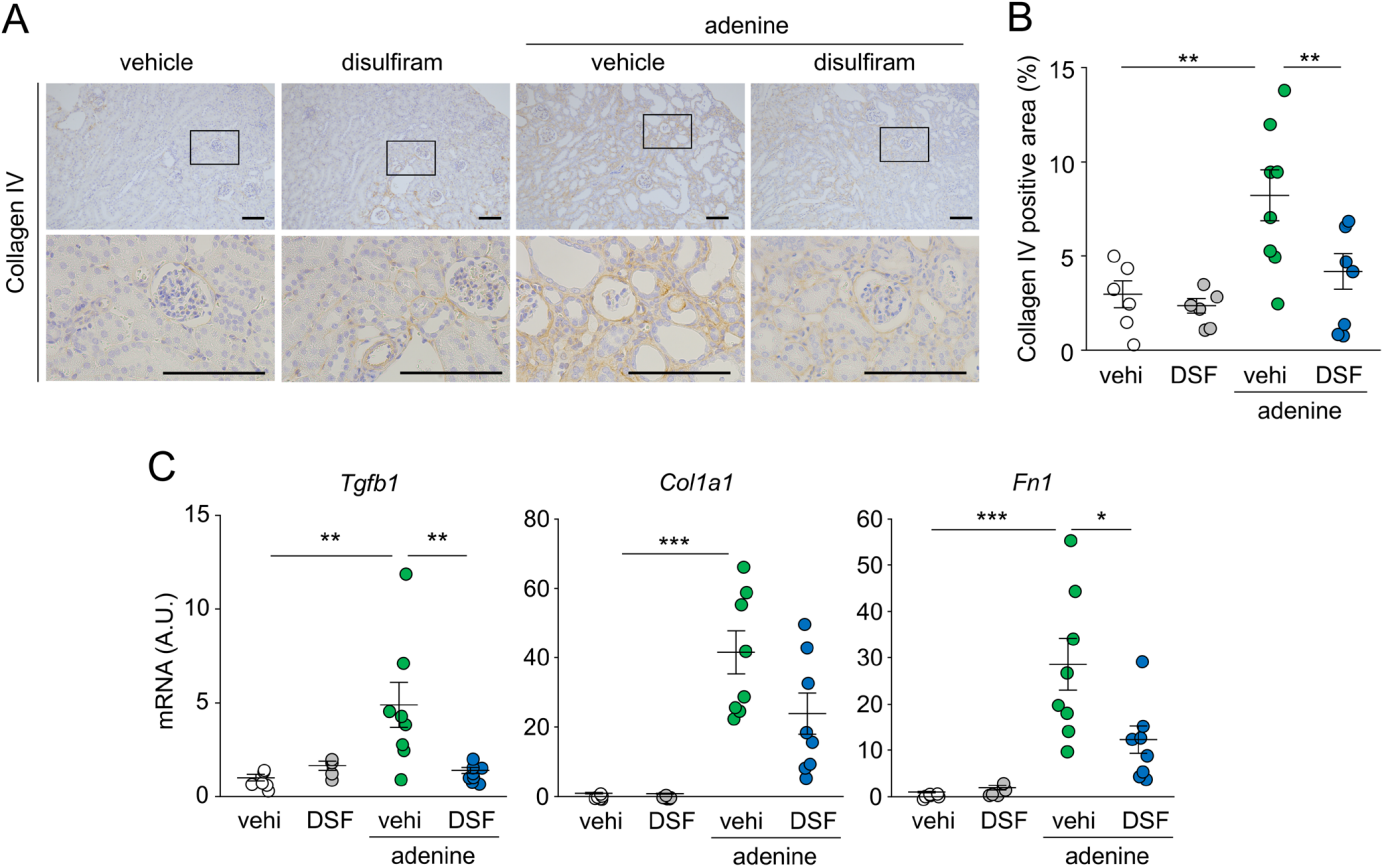
DSF alleviates renal fibrosis in adenine‐induced kidney injury. (A) Representative photomicrographs of collagen IV staining of the kidney. The boxed area in the low‐magnification image (upper panel) indicates the region shown at higher magnification in the lower panel. Scale bar = 100 μm. (B) Comparison of collagen IV‐positive areas. (C) Gene expression of *Tgfb1*, *Col1a1*, and *Fn1* in the kidney. A.U., arbitrary units; DSF, disulfiram; vehi, vehicle. Data are presented as mean ± standard error of the mean. *n* = 5–8. *p < 0.05, **p < 0.01, ***p < 0.001.

FAO disruption and accumulation of cellular senescence ultimately contribute to renal fibrosis [24, 25]. Immunohistochemical and gene expression analyses were performed to evaluate the effects of DSF on renal fibrosis. Immunohistochemical staining for collagen IV demonstrated that the collagen IV‐positive area, which increased in adenine‐induced nephropathy, was significantly attenuated by DSF treatment (Figure 5A,B). In parallel, renal mRNA expression levels of fibrosis‐related genes, including *Tgfb1*, *Col1a1*, and *Fn1*, were significantly elevated in adenine‐fed mice. Notably, the expression levels of *Tgfb1* and *Fn1* were significantly reduced following DSF administration (Figure 5C).

### 3.6 Adenine Crystal Formation in the Kidney

The renal injury observed in this model is driven by intratubular accumulation of 2,8‐dihydroxyadenine, an insoluble metabolite generated from adenine. This leads to inflammation and progressive tubulointerstitial fibrosis resulting in CKD [26]. To evaluate whether DSF treatment or food intake affected the process of adenine crystal formation, we quantified crystal deposition in the kidneys. DSF treatment did not significantly alter crystal burden at 2 weeks (*p* = 0.08), although there was a trend toward reduction. To further assess whether DSF influences early crystal formation, we shortened the administration period to 1 week and evaluated the mice at an earlier time point. Under these conditions, crystal deposition remained unchanged, whereas renal function parameters, including BUN and plasma cystatin C, were significantly improved (Figures S3 and S4).

### 3.7 DSF Failed to Repair Established Adenine‐Induced Nephropathy

**FIGURE 6:**
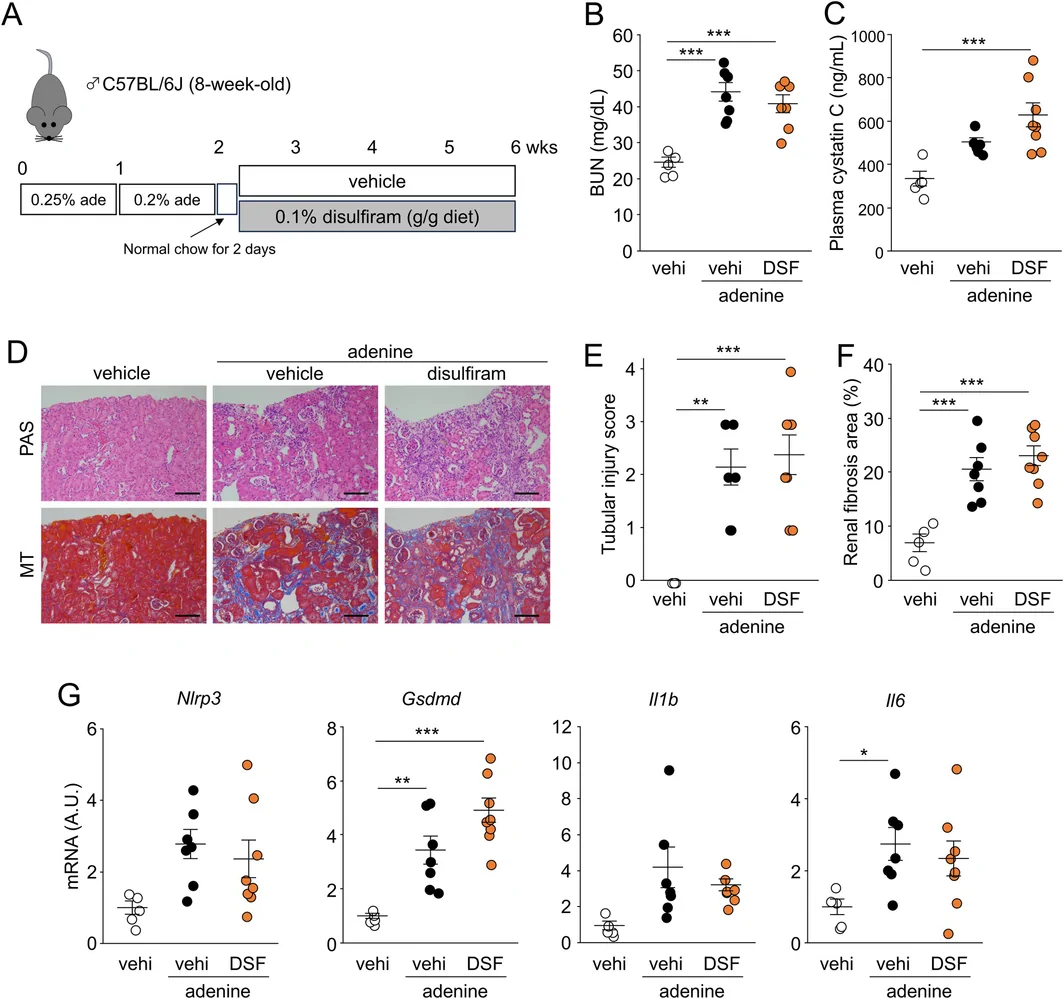
DSF fails to repair kidney injury after adenine treatment. (A) Experimental protocol. Plasma levels of blood urea nitrogen (BUN) (B) and cystatin C (C). (D) Representative photomicrographs of periodic acid–Schiff (PAS) and Masson's trichrome (MT) staining of the kidney. Scale bar = 100 μm. Comparison of tubular injury scores (E) and renal fibrosis area (F). (G) Gene expression of *Nlrp3*, *Gsdmd*, *Il1b*, and *Il6* in the kidney. A.U., arbitrary units; DSF, disulfiram; vehi, vehicle. Data are presented as mean ± standard error of the mean. *n* = 5–8. *p < 0.05, **p < 0.01, ***p < 0.001.

We further examined whether DSF promotes renal repair after the establishment of adenine‐induced nephropathy. The mice were fed an adenine‐containing diet for 2 weeks, followed by DSF administration for 4 weeks (Figure 6A). Plasma levels of BUN and cystatin C were similar between the vehicle and DSF groups (Figure 6B,C). Both tubular injury scores and renal fibrosis areas were unchanged by DSF treatment (Figure 6D–F). Finally, DSF did not affect the expression levels of *Nlrp3, Gsdmd, Il1b*, or *Il6* compared with those in the vehicle group with adenine‐induced nephropathy (Figure 6G).

### 3.8 GSDMD Expression Is Associated With Tubular Injury in Individuals With CKD

**FIGURE 7:**
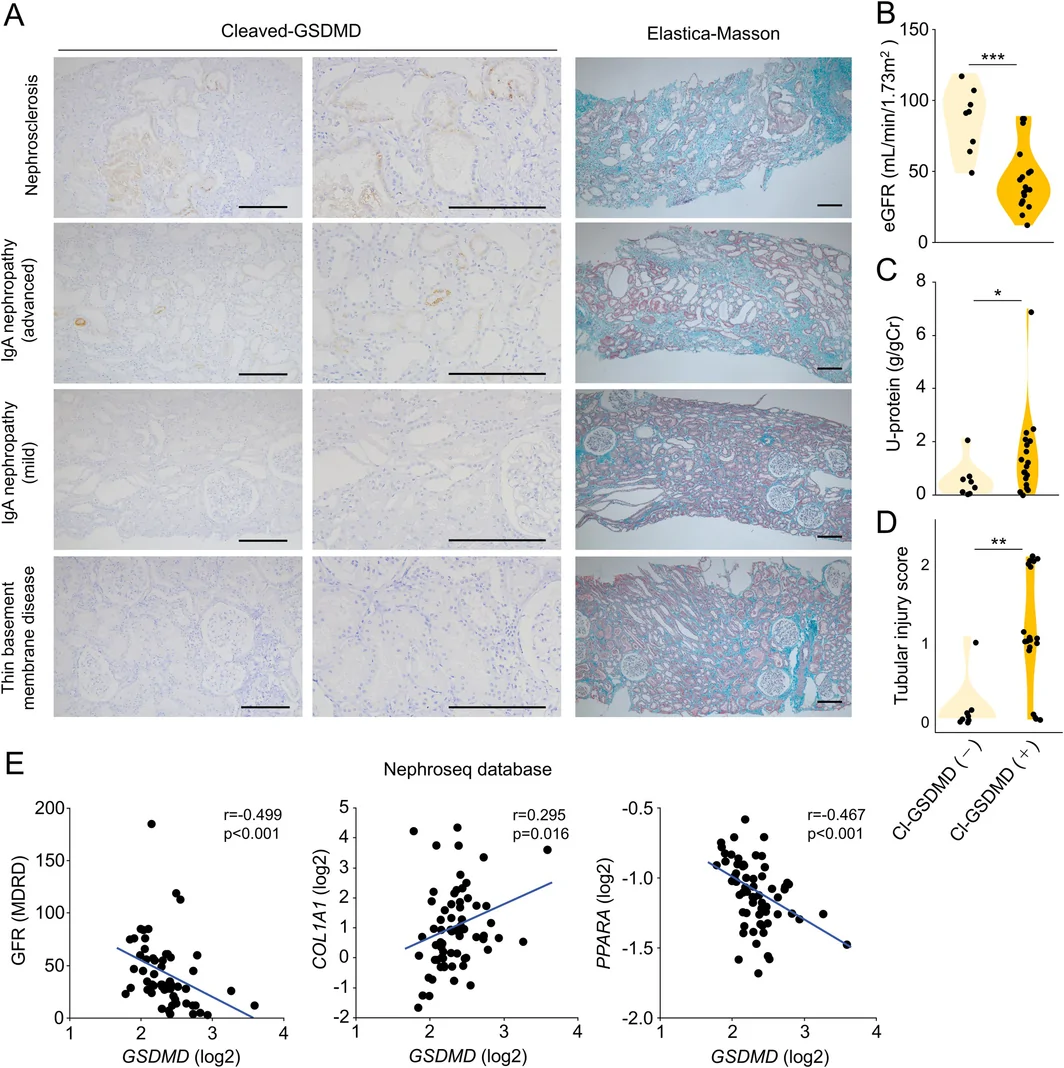
Expression of gasdermin D in individuals with CKD (A) Immunohistochemistry for cleaved gasdermin D (Cl‐GSDMD) and Elastica–Masson staining. The upper two cases represent advanced CKD corresponding to stage G4, whereas the lower two cases represent mild disease corresponding to stage G2. Scale bar = 200 μm. (B–D) Comparison of estimated glomerular filtration rate (eGFR), urinary protein (U‐protein), and tubular injury scores between Cl‐GSDMD‐positive and ‐negative groups in IgA nephropathy. (E) Correlation between renal *GSDMD* expression and GFR, *Col1A1*, and *PPARA* mRNA expression in transplanted kidneys. The data were obtained from the Nephroseq database. CKD, chronic kidney disease; gCr, grams of creatinine; GFR, glomerular filtration rate; MDRD, Modification of Diet in Renal Disease. *p < 0.05, **p < 0.01, ***p < 0.001.

GSDMD, a key mediator of pyroptosis, is one of the main targets of DSF [13, 14]. To gain further insights, we examined *GSDMD* expression in individuals with CKD. Using renal biopsy specimens obtained at our institution, we performed immunohistochemical staining for cleaved GSDMD, a marker of *GSDMD* activation. As shown in Figure 7A, strong tubular expression was observed in patients with impaired renal function and advanced fibrosis. Focusing specifically on IgA nephropathy, thereby minimizing heterogeneity among different kidney diseases, cleaved GSDMD positivity showed a stronger association with reduced eGFR and greater tubular injury severity (Table S3 and Figure 7B–D). Furthermore, using the Saint‐Mézard kidney transplant dataset in the public database Nephroseq, *GSDMD* expression negatively correlated with eGFR and *PPARA* expression, while showing a positive correlation with *COL1A1* expression, suggesting an association between increased *GSDMD* expression, CKD severity, and impairment of FAO‐related pathways (Figure 7E).

## 4 Discussion

In this study, we demonstrated that (1) DSF alleviated kidney injury in adenine‐induced nephropathy by reducing inflammatory cell infiltration and the expression of innate immune‐related genes; (2) RNA‐seq revealed a restoration of fatty acid metabolism in injured kidneys; (3) markers of DNA damage response, cellular senescence, and fibrosis were subsequently ameliorated by DSF; and (4) the expression of cleaved GSDMD or *GSDMD* correlated with disease severity in renal biopsy samples from patients with various underlying etiologies.

DSF is an established therapeutic agent for alcohol dependence; however, recent studies have revealed that it modifies a cysteine residue essential for GSDMD activation, thereby inhibiting pyroptosis [14]. Animal studies using AKI or renal fibrosis models have demonstrated that amelioration of kidney injury by DSF is associated with reduced *GSDMD* activation [17, 18, 19]. Additionally, DSF directly targets MD‐2, a co‐receptor involved in TLR4 activation, as well as NLRP3, a key inflammasome component, thereby suppressing multiple molecules associated with innate immune responses and exerting potent anti‐inflammatory effects [15, 16]. Therefore, we characterized the anti‐inflammatory effects of DSF, focusing on its ability to reduce macrophage and neutrophil infiltration and suppress innate immunity‐related gene expression in the kidney.

Innate immune activation reshapes mitochondrial function and FAO, while metabolic status regulates immune responses, a bidirectional interaction that has recently attracted attention in immunometabolism [27]. Disruption of this balance promotes persistent inflammation, impaired repair, and CKD progression [23, 28]. Innate immune activation induces maladaptive metabolic rewiring in tubular epithelial cells, thereby linking immune dysregulation to metabolic dysfunction and subsequent kidney injury [23]. Specifically, TLRs expressed on tubular cells recognize damage‐associated molecular patterns, a process that promotes renal fibrosis in animal models [29, 30]. Additionally, lipopolysaccharide, a canonical agonist of TLR4, suppresses FAO in the kidney [31]. Moreover, another study demonstrated that CD36 knockdown, a class B scavenger receptor, suppresses NLRP3 activation, IL‐1β expression, and reactive oxygen species production in tubular cells, while increasing the expression of FAO‐related enzymes and attenuating renal fibrosis in a diabetic kidney disease (DKD) model [32]. Collectively, these findings suggest a close link between innate immunity and metabolic responses in CKD, and that DSF may alleviate renal fibrosis by restoring FAO through the suppression of inflammatory responses.

Dysregulated FAO promotes cellular senescence under various pathological conditions, including cancer, hepatic steatosis, and CKD, thereby contributing to organ fibrosis [33, 34, 35, 36]. In the kidney, impaired FAO contributes to lipid accumulation, tubular dysfunction, and fibrosis, whereas PPARα‐mediated enhancement of FAO alleviates age‐related lipid accumulation and fibrotic changes in renal epithelial cells [36]. Additionally, dysregulated FAO promotes renal fibrosis, which is the final common pathway leading to end‐stage kidney disease. Restoration of fatty acid metabolism, either genetically (*Ppargc1a* transgenic mice) or pharmacologically (fenofibrate), protects mice from tubulointerstitial fibrosis [24]. Moreover, renal tubular *Cpt1a* overexpression protects against kidney fibrosis by restoring mitochondrial homeostasis in murine models of renal fibrosis [37]. Therefore, DSF‐dependent anti‐inflammatory effects, together with FAO restoration, may subsequently suppress cellular senescence and mitigate fibrotic responses in adenine‐induced nephropathy.

DSF treatment significantly improved renal function in adenine‐induced nephropathy. Because DSF‐treated mice exhibited increased food intake, we considered the possibility that altered adenine exposure might have affected crystal formation. However, at 1 week, crystal deposition did not differ between groups despite significant improvement in BUN, suggesting that the early renoprotective effects of DSF were independent of crystal burden. At 2 weeks, crystal deposition showed a nonsignificant tendency toward reduction (*p* = 0.08). Taken together, these findings indicate that the beneficial effects of DSF are unlikely to be solely explained by changes in adenine‐induced crystal formation and may instead involve modulation of downstream inflammatory responses.

Our results show that DSF ameliorates renal injury when administered during the progression phase of adenine‐induced nephropathy, but does not improve renal dysfunction once severe injury is established. This suggests that DSF mainly suppresses ongoing inflammation and slows disease progression, but cannot reverse established tubulointerstitial fibrosis. This interpretation is consistent with previous studies showing that DSF protects against cisplatin‐induced and ischemia–reperfusion‐induced AKI by preventing or limiting the development of renal injury [17, 38]. Therefore, DSF may act as a disease‐modifying therapy rather than as a regenerative therapy for CKD. Further studies adjusting the timing of intervention or extending treatment duration are needed to determine whether partial functional recovery is possible after injury has developed.

In individuals with CKD, we demonstrated a correlation between *GSDMD* expression, a key molecular target of DSF, and clinical disease severity, and further showed that *GSDMD* expression increases in association with renal fibrosis, suggesting the clinical relevance of this molecule. Previous studies have reported increased *GSDMD* expression in inflammatory cells such as macrophages and tubular epithelial cells [39, 40, 41, 42]. In mouse models of angiotensin II‐induced kidney injury and DKD, *GSDMD* expression was increased in renal tubules, and similar changes were observed in kidney biopsy specimens from patients with DKD [39, 40]. Additionally, single‐cell transcriptomic analysis in a nephrocalcinosis model demonstrated increased *GSDMD* expression in injured tubular cells [41]. Notably, our study identified the localization of cleaved GSDMD (activated form of the protein) within the kidney, enabling us to determine the cells in which GSDMD is active and provide insights into its functional role in tubular epithelial cells.

This study has several limitations. We primarily focused on the anti‐inflammatory effects of DSF in ameliorating adenine‐induced nephropathy. Nevertheless, DSF exerts diverse therapeutic actions, and the contribution of mechanisms other than anti‐inflammatory effects cannot be excluded. Whether GSDMD mediates the renoprotective effects of DSF remains unclear, and comparison with GSDMD‐deficient mice would help clarify this issue. Notably, ALDH2 suppresses renal fibrosis [43]; however, the extent to which the original pharmacological action of DSF, ALDH2 inhibition, contributes to these effects remains unclear. Moreover, dysregulation of the gut microbiota is involved in CKD pathogenesis [44], and DSF modulates the gut microbiota to ameliorate nonalcoholic steatohepatitis and atherosclerosis [45, 46]. Additionally, our human study evaluated only the association between GSDMD expression and renal impairment; therefore, a causal relationship could not be established.

Collectively, our findings demonstrate that DSF alleviates renal fibrosis by modulating inflammatory and immunometabolic responses. Given that DSF is already approved by the Food and Drug Administration and has an established safety profile, these findings support further preclinical and clinical studies to evaluate the potential of DSF for CKD.

## Author Contributions

S.K. and Y.O. conceived and designed animal study. Y.O. conceived and designed human study. S.K., Y.O., S.I., and I.F. performed experiments and analyzed data. S.K., Y.O., S.I., I.F., M.M., and T.T. interpreted results of experiments. S.K. and Y.O. drafted manuscript and prepared figures. S.I., I.F., M.M. and T.T. edited and revised manuscript.

## Funding

This work was supported by MEXT|Japan Society for the Promotion of Science (JSPS), 21K16156; Eli Lilly Japan K.K. Innovation Research Grand 2024; Koyanagi Foundation; Japan Kidney Foundation; Miyagi Kidney Foundation.

## Conflicts of Interest

The authors declare no conflicts of interest.

## Supporting information


**Table S1:** Primer sequence.
**Table S2:** Characteristics of the mice at sacrifice in protocol 1.
**Table S3:** Basal characteristics of the patients with IgA nephropathy.
**Figure S1:** RNA‐seq analysis comparing between control and adenine‐treated mice. (A) Volcano plot. (B, C) KEGG enrichment analysis of differentially expressed genes between vehicle‐ and DSF‐treated control mice. The top 10 up‐ and downregulated pathways are shown n=3 per group.
**Figure S2:** RNA‐seq analysis comparing vehicle and DSF treatment in adenine‐treated mice.(A) Volcano plot. (B) Heatmap of differentially expressed genes n=3 per group.
**Figure S3:** Adenine crystal formation after 2 weeks of adenine treatment. (A) Photomicrographs of hematoxylin and eosin (HE) staining. Arrowheads indicate adenine crystals. Scale bar =100μm. (B) Comparison of the number of adenine crystals in the kidney. DSF, disulfiram; hpf, high power field; n.s, not significant; vehi, vehicle; Data are presented as mean ± standard error of the mean. n=6−8.
**Figure S4:** Adenine crystal formation after 1 week of adenine treatment. (A) Experimental protocol. (B) Plasma levels of blood urea nitrogen. (C) Plasma levels of cystatin C. (D) Photomicrographs of hematoxylin and eosin (HE) staining. Arrowheads indicate adenine crystals. Scale bar = 100 μm. (E) Comparison of the number of adenine crystals in the kidney. ade, adenine; DSF, disulfiram; hpf, high power field; n.s, not significant. Data are presented as mean ± standard error of the mean. *n* = 6. *p < 0.05, **p < 0.01.

## Data Availability

All data supporting the findings of this study are available within the article. Additional raw data are available from the corresponding author upon request. The RNA‐seq data generated in this study have been deposited in the DNA Data Bank of Japan under BioProject accession number PRJDB42838.
