# Supplementary material for: Disulfiram Attenuates Adenine‐Induced Renal Fibrosis by Modulating Inflammatory and Immunometabolic Responses
Source: FASEB J. 2026 Sep 17;40(18):e72299. doi: 10.1096/fj.202601271RR (PMC13583549; doi:10.1096/fj.202601271RR)
Supplement: Supplementary file 1 [file FSB2-40-e72299-s001.pdf]

Supplementary Tables

Supplementary Table 1. Primer sequence

Gene	sequence (5'→3')	Gene	sequence (5'→3')
Nlrp3	Fw: TGTGAGAAGCAGGTTCTACTCT	*p53*	Fw: CTCTCCCCCGCAAAAGAAAAA
	Rv: TGTAGCGACTGTTGAGGTCCA		Rv: CGGAACATCTCGAAGCGTTTA
Gsdmd	Fw: TTCAGGCCCTACTGCCTTCT	*p21*	Fw: TCCACAGCGATATCCAGACA
	Rv: GTTGACACATGAATAACGGGGTT		Rv: GGACATCACCAGGATTGGAC
Il1b	Fw: CTGTGACTCATGGGATGATGATG	*p16*	Fw: TGTTGAGGCTAGAGAGGATCTTG
	Rv: CGGAGCCTGTAGTGCAGTTG		Rv: CGAATCTGCACCGTAGTTGAGC
Il6	Fw: TAGTCCTTCCTACCCCAATTTCC	*Tgfb1*	Fw: CTCCCGTGGCTTCTAGTGC
	Rv: TTGGTCCTTAGCCACTCCTTC		Rv: GCCTTAGTTTGGACAGGATCTG
Tnfa	Fw: CCCTCACACTCAGATCATCTTCT	*Col1a1*	Fw: GCTCCTCTTAGGGGCCACT
	Rv: GCTACGACGTGGGCTACAG		Rv: CCACGTCTCACCATTGGGG
Acox1	Fw: CTTGGATGGTAGTCCGGAGA	*Fn1*	Fw: ACAAGGTTCGGGAAGAGGTT
	Rv: TGGCTTCGAGTGAGGAAGTT		Rv: CCGTGTAAGGGTCAAAGCAT
Acox2	Fw: TACCAACGCCTGTTTGAGTG	*Hprt*	Fw: TCAGTCAACGGGGGACATAAA
	Rv: TTTCCAGCTTTGCATCAGTG		Rv: GGGGCTGTACTGCTTAACCAG
Ppara	Fw: TGCAAACTTGGACTTGAACG	*Ppargc1a*	Catalog ID: MA244883 (Takara Bio)
	Rv: GATCAGCATCCCGTCTTTGT		

Abbreviations: Fw, forward; Rv, reverse

References:

1. Kin, S. et al. Nicotinamide Attenuates Complement and Coagulation Pathways and Resultant Renal Fibrosis. FASEB J 39, e71263 (2025). https://doi.org/10.1096/fj.202502028R
2. Al-Dabet, M. M. et al. Reversal of the renal hyperglycemic memory in diabetic kidney disease by targeting sustained tubular p21 expression. Nat Commun 13, 5062 (2022). https://doi.org/10.1038/s41467-022-32477-9
3. Kang, H. M. et al. Defective fatty acid oxidation in renal tubular epithelial cells has a key role in kidney fibrosis development. Nat Med 21, 37-46 (2015). https://doi.org/10.1038/nm.3762
4. Primer Bank (http://pga.mgh.harvard.edu/primerbank/)
5. Takara Bio Inc. (Shiga, Japan)

Supplementary Table 2. Characteristics of the mice at sacrifice in protocol 1

	Control vehicle	Control DSF	Adenine vehicle	Adenine DSF
Body wt (g)	23.1 ± 0.4	22.1 ± 0.4	15.6 ± 0.4[a]	17.1 ± 0.3[a]
Rt Kidney wt/BW (mg/g)	6.6 ± 0.3	6.3 ± 0.2	9.3 ± 0.4[a]	7.1 ± 0.3[b]
Lt Kidney wt/BW (mg/g)	6.3 ± 0.3	6.3 ± 0.2	8.7 ± 1.5[a]	6.5 ± 0.2[b]
Liver wt (mg/g)	46.7 ± 1.6	47.7 ± 0.7	46.3 ± 0.5	41.4 ± 0.5[a,b]
Food intake (g/cage/day)*				
1st week	5.0 ± 0.3	4.6 ± 0.1	3.0 ± 0.2[a]	3.6 ± 0.1[a]
2nd week	5.1 ± 0.2	4.1 ± 0.2[a]	2.6 ± 0.2[a]	4.0 ± 0.2[b]

Abbreviations: Rt, right; Lt, left; BW, body weight; wt, weight. n = 6-8

Data are presented as mean ± standard error of the mean.

*Food intake was measured per cage (two mice per cage, n = 3-4).

a, $p<0.05$ vs control + vehicle; b, $p<0.05$ vs adenine + vehicle.

Supplementary Table 3. Basal characteristics of the patients with IgA nephropathy

	Cl-GSDMD negative (n = 8)	Cl-GSDMD positive (n = 19)	p-value
Sex (M/F)	3/5	7/12	n.s.
Age	31.4 ± 14.9	49.4 ± 16.0	p=0.02
BMI (kg/m^2)	20.8 ± 1.8	25.0 ± 3.7	p=0.005
Serum creatinine (mg/dL)	0.76 ± 0.21	1.46 ± 0.17	p=0.02
eGFR (ml/min/1.73 m^2)	92.5 (66.8-105.5)	38 (29-51)	p<0.001
U-protein (g/g creatinine)	0.44 (0.12-0.72)	1.12 (0.44-2.08)	p=0.049
Oxford classification			
M0	3/8 (37.5%)	6/19 (31.6%)	n.s.
M1	5/8 (62.5%)	13/19 (68.4%)	
E0	6/8 (75%)	15/19 (78.9%)	n.s.
E1	2/8 (25%)	4/19 (21.1%)	
S0	1/8 (12.5%)	8/19 (42.1%)	n.s.
S1	7/8 (87.5%)	11/19 (57.9%)	
T0	7/8 (87.5%)	4/19 (21.1%)	p=0.005
T1	1/8 (12.5%)	9/19 (47.4%)	
T2	0/8 (0%)	6/19 (31.6%)	
C0	6/8 (75%)	17/19 (89.5%)	n.s.
C1	2/8 (25%)	2/19 (10.5%)	
C2	0/8 (0%)	0/19 (0%)	

Abbreviations: Cl-GSDMD, cleaved-gasdermin D; M, male; F, female; BMI, body mass index; eGFR, estimated glomerular filtration rate; U-protein, urinary protein; n.s., not significant.
Data are presented as mean ± standard deviation and median with interquartile range for normally and non-normally distributed variables, respectively.

Supplementary Figures

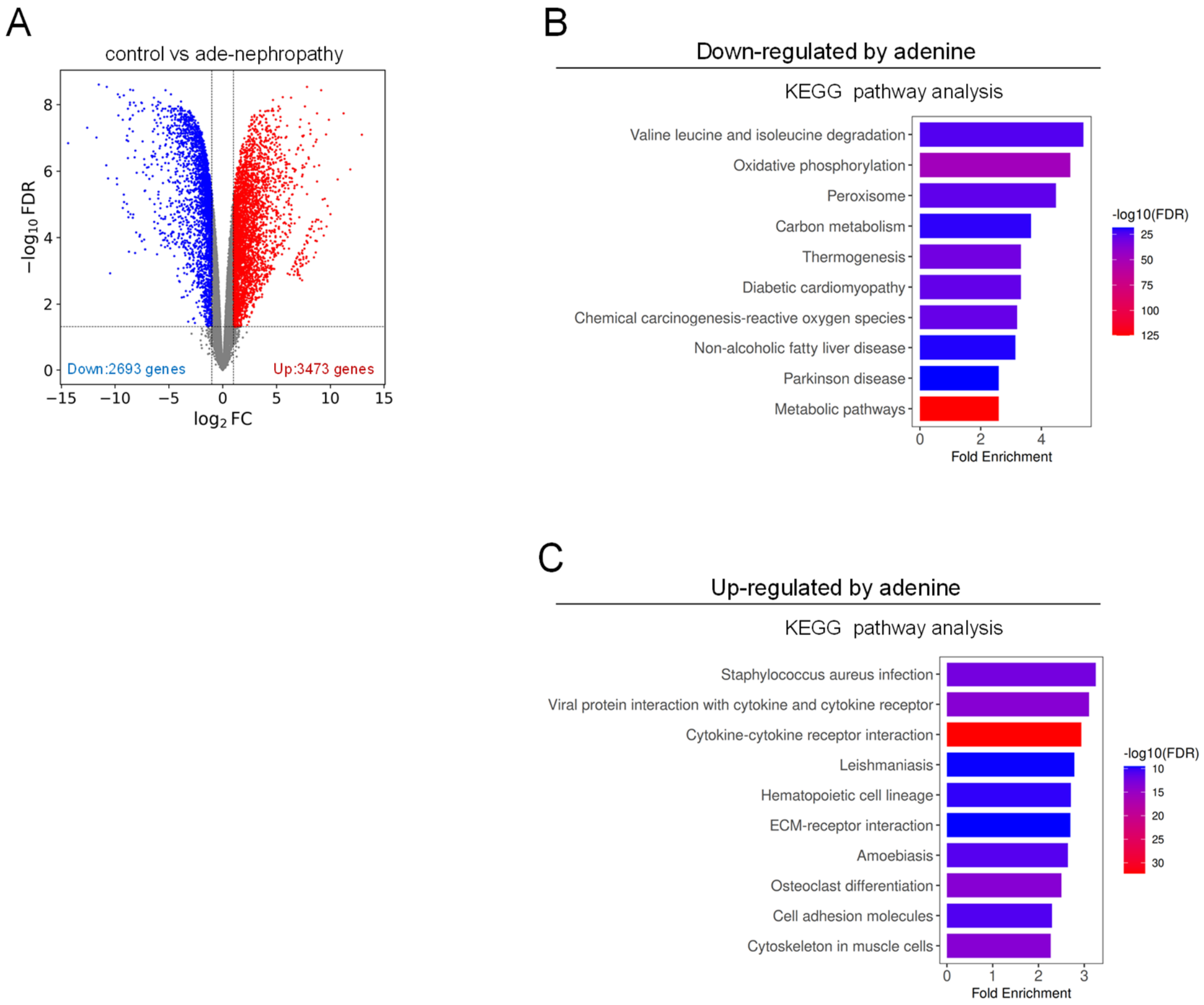

Supplementary Figure 1. RNA-seq analysis comparing between control and adenine-treated mice.

(A) Volcano plot. (B–C) KEGG enrichment analysis of differentially expressed genes between vehicle- and DSF-treated control mice. The top 10 up- and downregulated pathways are shown. n = 3 per group.

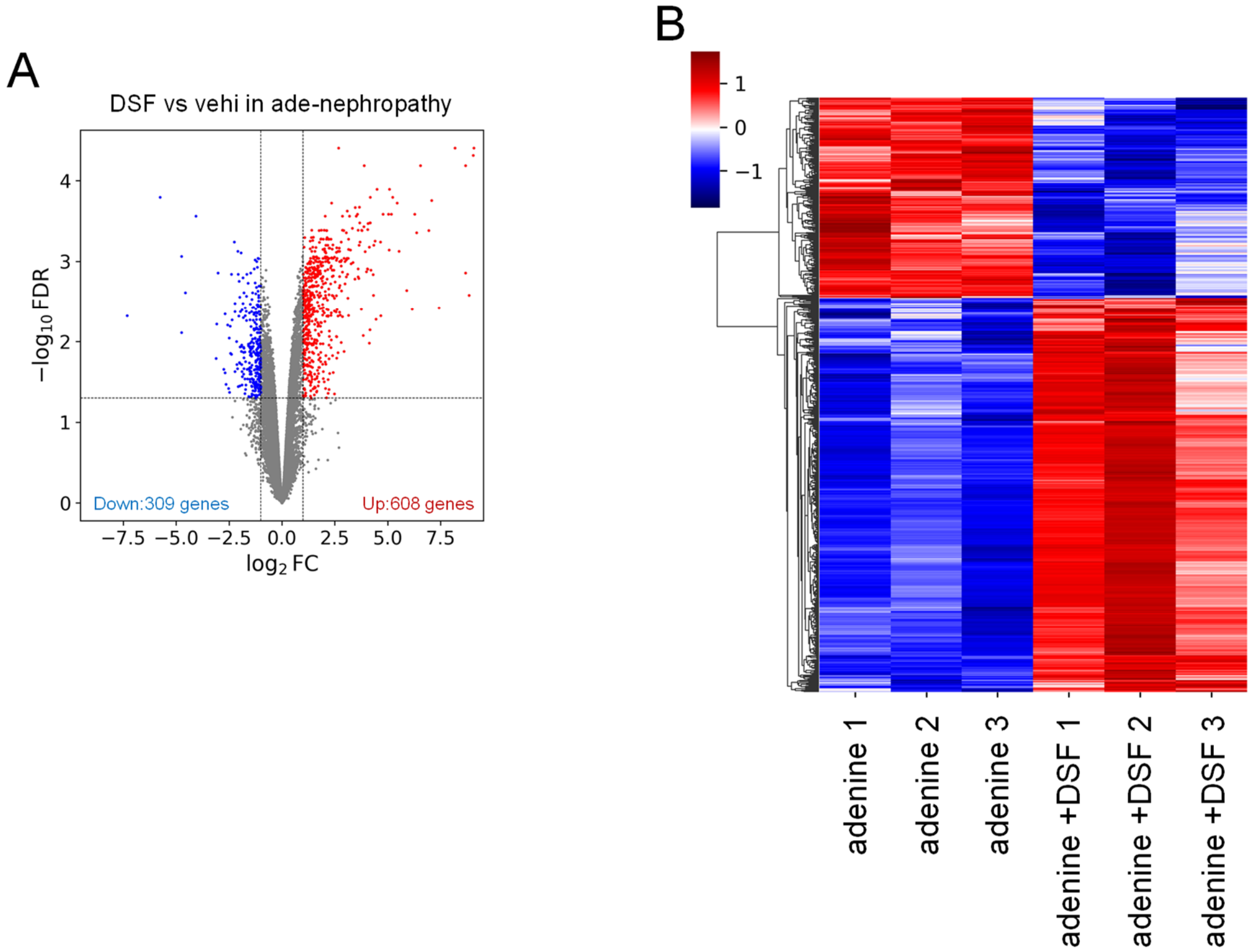

Supplementary Figure 2. RNA-seq analysis comparing vehicle and DSF treatment in adenine-treated mice.

(A) Volcano plot. (B) Heatmap of differentially expressed genes. n = 3 per group.

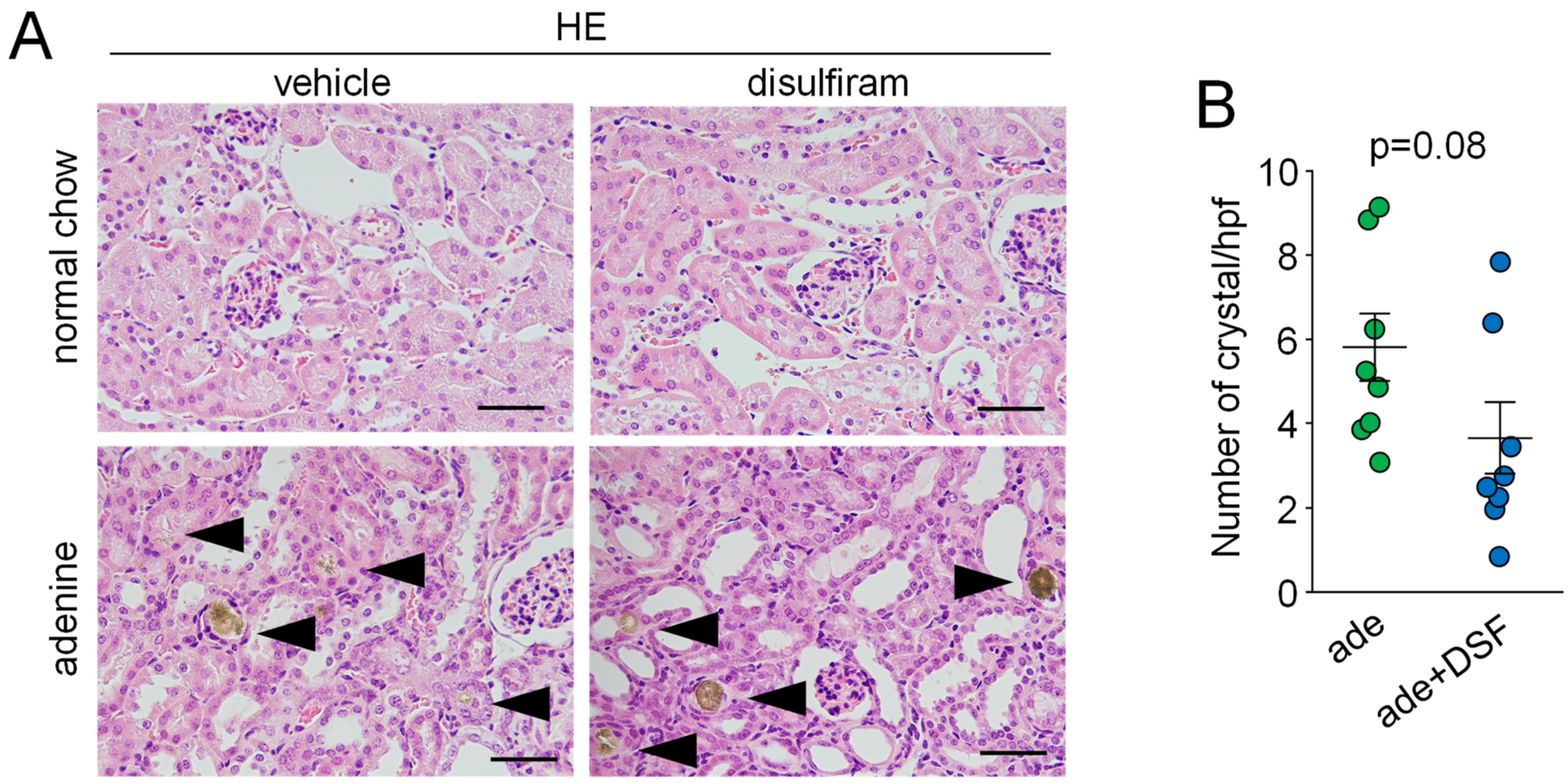

Supplementary Figure 3. Adenine crystal formation after two weeks of adenine treatment.

(A) Photomicrographs of hematoxylin and eosin (HE) staining. Arrowheads indicate adenine crystals. Scale bar = 100 μm. (B) Comparison of the number of adenine crystals in the kidney. vehi, vehicle; DSF, disulfiram; n.s, not significant; hpf, high power field. Data are presented as mean ± standard error of the mean. n = 6–8.

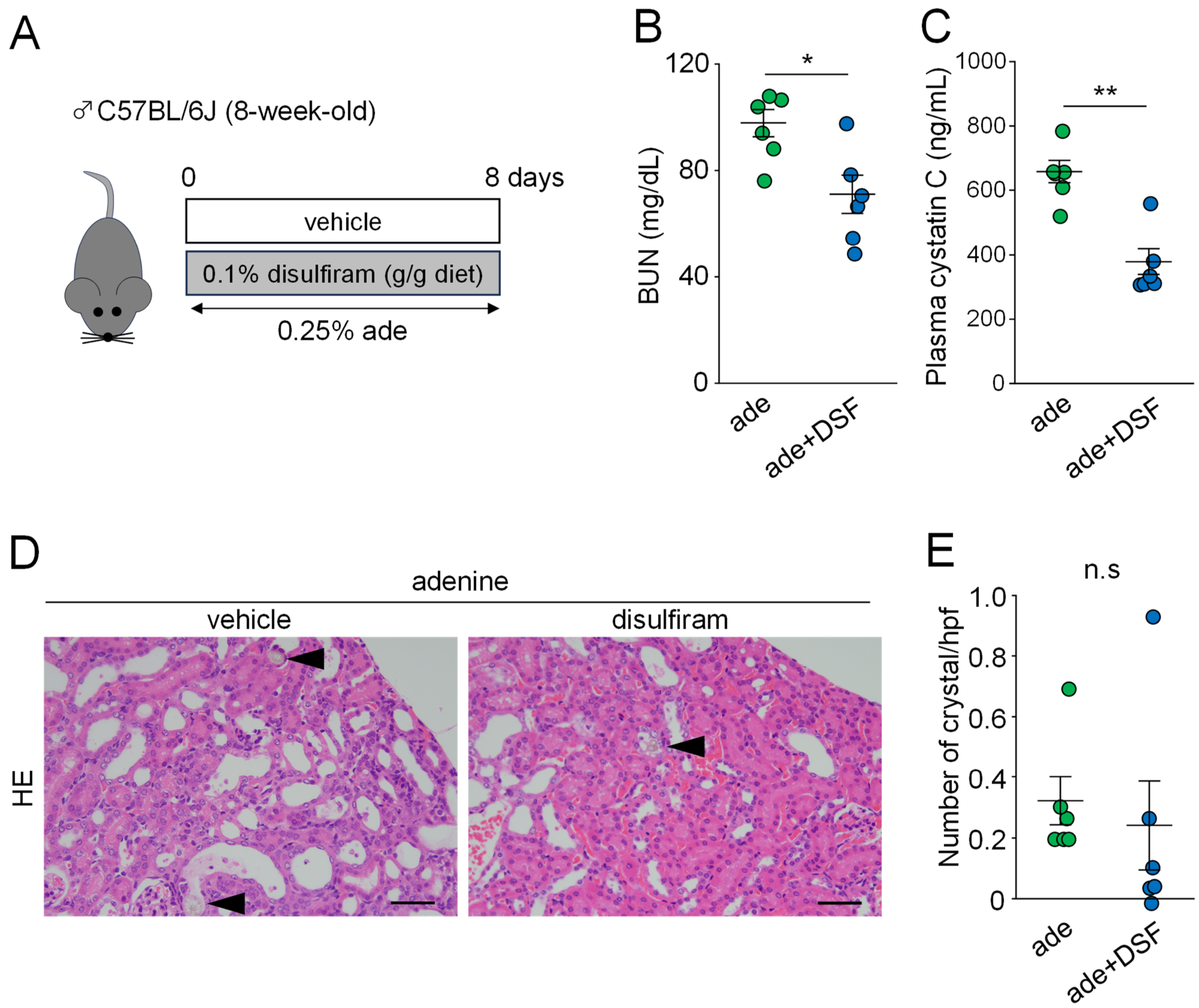

Supplementary Figure 4. Adenine crystal formation after one week of adenine treatment.

(A) Experimental protocol. (B) Plasma levels of blood urea nitrogen. (C) Plasma levels of cystatin C. (D) Photomicrographs of hematoxylin and eosin (HE) staining. Arrowheads indicate adenine crystals. Scale bar = 100 μm. (E) Comparison of the number of adenine crystals in the kidney. ade, adenine; DSF, disulfiram; n.s, not significant; hpf, high power field. Data are presented as mean ± standard error of the mean. n = 6. *p<0.05, **p<0.01
